# Supplementary material for: Perforated Hydrogels Consisting of Cholesterol-Bearing Pullulan (CHP) Nanogels: A Newly Designed Scaffold for Bone Regeneration Induced by RANKL-Binding Peptides and BMP-2
Source: Int J Mol Sci. 2022 Jul 14;23(14):7768. doi: 10.3390/ijms23147768 (PMC9316061; doi:10.3390/ijms23147768)
Supplement: Supplementary file 1 [file ijms-23-07768-s001.zip › ijms-1775303-supplementary.pdf]

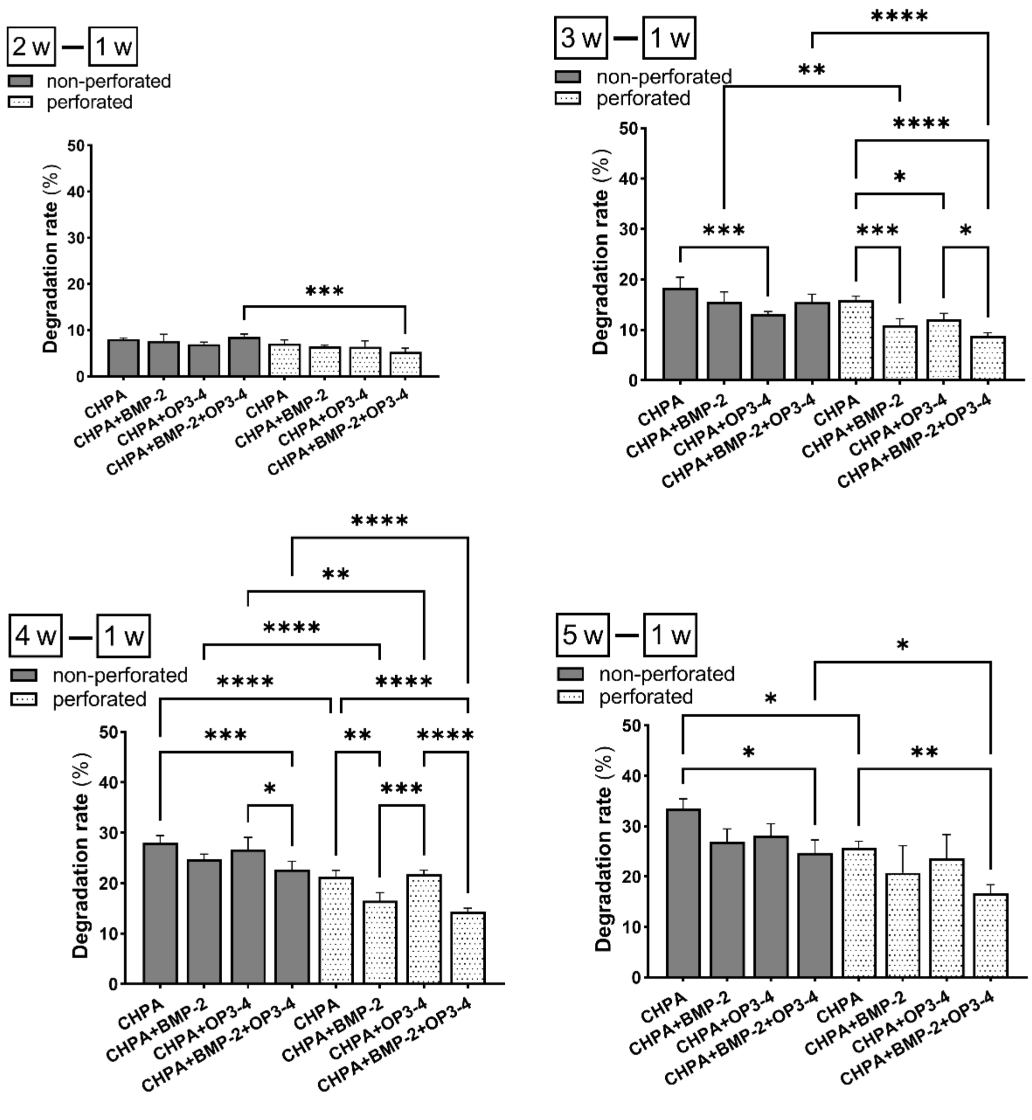

Supplementary Figure S1. The time-course analyses of the degradation rate of CHPA scaffold. Quantitative data of the degradation rate of the two differently designed scaffolds. The percentage reduction of scaffold material was calculated by taking the fluorescent area at week 1 as 100% and subtracting the fluorescent area percentage every week and calculated as described in the Materials and Methods. See the Figure 2 legends regarding the group names. The comparisons between groups were analyzed by the ANOVA and Tukey HSD test. Values are expressed as the mean ± SD, *$p < 0.05$, **$p < 0.01$, ***$p < 0.001$, ****$p < 0.0001$.

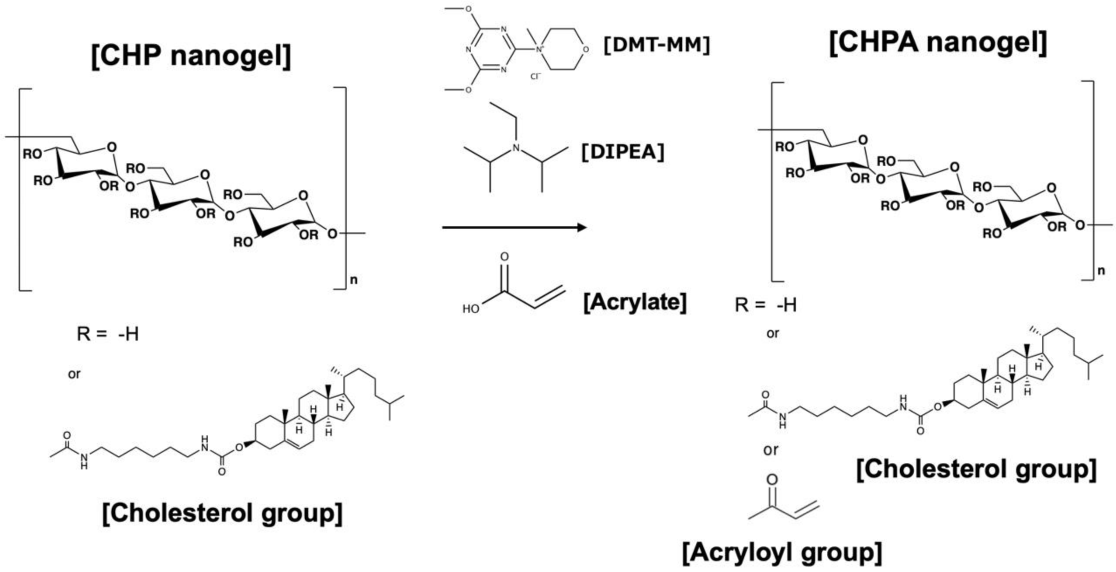

Supplementary Figure S2. Synthesis route of CHPA nanogels.

CHP: cholesterol-bearing pullulan nanogel

DMT-MM: 4-(4,6-dimethoxy-1,3,5-triazin-2-yl)-4-methyl-morpholinium chloride

DIPEA: N,N-diisopropylethylamine, and acrylic acid
